# High Serum Advanced Glycation End Products Are Associated with Decreased Insulin Secretion in Patients with Type 2 Diabetes: A Brief Report

**DOI:** 10.1155/2017/5139750

**Published:** 2017-06-12

**Authors:** Tsuyoshi Okura, Etsuko Ueta, Risa Nakamura, Yohei Fujioka, Keisuke Sumi, Kazuhisa Matsumoto, Kyoko Shoji, Kazuhiko Matsuzawa, Shoichiro Izawa, Yuri Nomi, Hitomi Mihara, Yuzuru Otsuka, Masahiko Kato, Shin-ichi Taniguchi, Kazuhiro Yamamoto

**Affiliations:** ^1^Division of Cardiovascular Medicine, Endocrinology and Metabolism, Department of Molecular Medicine and Therapeutics, Tottori University Faculty of Medicine, Yonago, Tottori, Japan; ^2^School of Health Science, Tottori University Faculty of Medicine, Yonago, Tottori, Japan; ^3^Department of Regional Medicine, Tottori University Faculty of Medicine, Yonago, Tottori, Japan; ^4^Faculty of Applied Life Sciences, Niigata University of Pharmacy and Applied Life Sciences, Niigata, Japan; ^5^Department of Food and Nutrition, Toita Women's College, Tokyo, Japan

## Abstract

**Objective:**

Advanced glycation end products (AGEs) are important in the pathophysiology of type 2 diabetes mellitus (T2DM). They directly cause insulin secretory defects in animal and cell culture models and may promote insulin resistance in nondiabetic subjects. We have developed a highly sensitive liquid chromatography-tandem mass spectrometry method for measuring AGEs in human serum. Here, we use this method to investigate the relationship between AGEs and insulin secretion and resistance in patients with T2DM.

**Methods:**

Our study involved 15 participants with T2DM not on medication and 20 nondiabetic healthy participants. We measured the AGE carboxyethyllysine (CEL), carboxymethyllysine (CML), and methyl-glyoxal-hydro-imidazolone (MG-H1). Plasma glucose and insulin were measured in these participants during a meal tolerance test, and the glucose disposal rate was measured during a euglycemic-hyperinsulinemic clamp.

**Results:**

CML and CEL levels were significantly higher in T2DM than non-DM participants. CML showed a significant negative correlation with insulin secretion, HOMA-%B, and a significant positive correlation with the insulin sensitivity index in T2DM participants. There was no correlation between any of the AGEs measured and glucose disposal rate.

**Conclusions:**

These results suggest that AGE might play a role in the development or prediction of insulin secretory defects in type 2 diabetes.

## 1. Introduction

Type 2 diabetes mellitus is a heterogeneous disease characterized by insulin resistance and defective insulin secretion [1]. Advanced glycation end products (AGEs) are produced by a nonenzymatic reaction between amino and carbonyl groups [2]. This reaction, called the Maillard or amino-carbonyl reaction, is accelerated in the state of hyperglycemia in diabetes. AGEs have been reported to be correlated with the progression of diabetes and aging [3]. An animal study demonstrated that exposure to excess AGEs activates pathways of *β*-cell damage which, via mitochondrial superoxide generation, can impair insulin secretion [4]. A human study also showed a cross-sectional association between AGEs and acute insulin secretion during glucose tolerance testing in healthy humans [5]. Another human study demonstrated that the circulating level of AGEs is associated with insulin resistance as evaluated by the homeostasis model assessment for insulin resistance (HOMA-IR), even in nonobese, nondiabetic subjects [6]. These results suggest that AGEs may affect insulin secretion as well as insulin resistance. However, there are few studies on the relationship between AGEs and insulin secretion ability or insulin resistance in patients with type 2 diabetes. Moreover, the most precise method for assessing insulin resistance is the glucose clamp technique, but this method is very complicated [7]. Instead, the HOMA-IR index is widely used in clinical practice and in clinical studies [8]. However, the validity of HOMA-IR may be limited in some patients, particularly those with a low BMI, reduced *β*-cell function, and high fasting glucose levels [9]. Since Asian and Japanese patients often show reduced *β*-cell function [10], a clamp study is required for evaluating insulin resistance in these populations.

N*ε*-(carboxymethyl)lysine (CML), N*ε*-(carboxyethyl)lysine (CEL), glyoxal-derived hydroimidazolone (G-H1), and methylglyoxal-derived hydroimidazolone (MG-H1) are known as representative AGEs generated in vivo [2]. Some AGEs such as CML are formed by an oxidative process and are called glycoxidation products. Several methods have been developed to determine the AGE content in biological samples. CEL, CML, and pentosidine have been measured using an immunological method with antibodies like rabbit-anti-CML-IgG and D12 antibody for CML [2]. Although ELISA is rapid, specific antibodies for each compound are required, the results are expressed in arbitrary units instead of actual concentrations, and the sample matrix, which can lead to incorrect estimation of AGE levels, affects the specificity of the assay significantly [11]. Recently, we developed a method for the simultaneous quantitation of several AGEs in brown-colored food using liquid chromatography-tandem mass spectrometry without ion-pair reagents and derivatization (LC-MS/MS method) [2]. We also developed a method for the simultaneous quantitation of several AGEs in human serum using LC-MS/MS method in this study.

According to past reports, there were some reports about the relationship between AGEs and insulin secretion and insulin resistance in the human healthy subjects; however, there were few reports about type 2 DM subjects. Furthermore, there were some reports about the relationship between AGEs and insulin secretion and insulin resistance by using the ELISA method; however, there were few reports about the exact quantitation of AGEs by using LC-MS/MS methods. Therefore, the aim of this study is to investigate the relationship between AGEs and insulin secretion and insulin resistance in the type 2 DM subjects by using LC-MS/MS methods. Based on previous studies, we hypothesized that patients with type 2 diabetes mellitus would show a correlation between AGE content and both insulin secretion ability and insulin resistance. In this study, we performed a meal tolerance test (MTT) and a glucose clamp in Japanese patients with type 2 diabetes mellitus and nondiabetic healthy volunteers and measured serum AGEs by the LC-MS/MS method.

## 2. Research Design and Methods

### 2.1. Subjects

Nine males and six females with type 2 diabetes mellitus (T2DM participants) participated in this study at Tottori University Hospital between 2014 and 2016. Type 2 diabetes mellitus was diagnosed using the criteria of the World Health Organization [12]. Patients with pancreatic disease, liver disease, or renal failure or those taking diabetogenic medications such as corticosteroids were excluded from this study. All T2DM participants were on diet therapy alone. Twelve male and eight female nondiabetic healthy volunteers (non-DM participants) were also recruited for this study. None of the non-DM participants had type 2 diabetes mellitus or were taking diabetic medications. Participant characteristics from the T2DM and non-DM groups are given in Table 1. The mean age, BMI, waist circumstance, fasting plasma glucose (FPG), HbA1c, and HOMA-IR of the DM group were significantly higher than those of the non-DM group, and the mean insulin sensitivity index (ISI) and glucose disposal rate (GDR) of the DM group were significantly lower than those of the non-DM group. There was no significant difference in insulin AUC and insulinogenic index (IGI) between the DM group and non-DM group. All participants were examined using the protocols reported in our previous study [13].

This study was approved by the Ethics Committee of the Faculty of Medicine, Tottori University (approval number G161). Informed consent was obtained from all of the participants using a procedure approved by the Ethics Committee.

### 2.2. Meal Tolerance Test

After fasting for at least 12 h, participants visited the clinic in the morning and consumed a test meal prepared by the Japan Diabetes Society (460 kcal/1882 kJ; 15% protein, 35% fat, and 50% carbohydrate; 1.6 g salt) [14]. Plasma glucose and insulin were measured at 0 (fasting), 30, 60, 120, and 180 min after the test meal. Plasma glucose was measured using the glucose oxidase method. Plasma insulin levels were measured using chemiluminescent immunoassays. Plasma insulin was defined as immunoreactive insulin (IRI). HbA1c was measured by high-performance liquid chromatography. HbA1c percentage values were converted to International Federation of Clinical Chemistry values (mmol/mol) using the HbA1c converter developed by the National Institutes of Diabetes and Digestive and Kidney Diseases [15].

### 2.3. Euglycemic-Hyperinsulinemic Clamp

Glucose clamps were performed 2 days after the MTT. We examined the participants in the morning after an overnight fast. We cannulated an antecubital vein to administer the infusate, and we also cannulated a dorsal vein and kept warm to facilitate venous sampling and provide arterialized venous blood. We performed the euglycemic-hyperinsulinemic clamp to determine insulin sensitivity in the peripheral tissues by using an artificial endocrine pancreas (STG 55; Nikkiso, Shizuoka, Japan) [7]. We used a primed constant infusion of insulin (100 mU/m^2^/min) and computer-controlled exogenous infusion of a glucose solution to achieve steady-state plasma insulin levels and maintain plasma glucose levels at 5.2 mmol/L (95 mg/dL). The previous studies reported that the steady-state plasma insulin level was 1200 pmol/L in patients with type 2 diabetes mellitus, by using this insulin infusion protocol [16, 17]. We calculated the steady-state glucose infusion rate between 90 and 120 min, and we defined the mean glucose infusion rate during this time as GDR (glucose disposal rate), which was used as a marker of peripheral insulin sensitivity. The glucose clamp method is a well-established procedure at our hospital [13, 18].

In a previous report, a GDR > 10.0 mg·kg^−1^·min^−1^ at an insulin infusion rate of 100 mU/m^2^/min was considered normal [19], and a GDR < 5.0 mg·kg^−1^·min^−1^ was considered to be obviously insulin resistant [20].

### 2.4. Calculation of Insulin Resistance and Secretion Indexes

HOMA-IR [8] = [fasting plasma glucose (mmol/L)] × [fasting plasma insulin (pmol/L)]/135. The normal range for HOMA-IR is <2.5 [21].

HOMA-%B (homeostasis model assessment of beta cell function) [8] = {20 × [fasting plasma insulin (pmol/L)]}/{[fasting plasma glucose (mmol/L)] − 3.5} (%).

Insulin sensitivity index (ISI) [22] = 10,000/√{[fasting plasma glucose (mmol/L) × fasting plasma insulin (pmol/L)] × [mean glucose × mean insulin during the MTT]}. The normal range for ISI is >2.5 [23].

Insulinogenic index (IGI) [24] = {[insulin (pmol/L) at 30 min] − [insulin (pmol/L) at 0 min]}/{[glucose (mmol/L) at 30 min] − [glucose (mmol/L) at 0 min]}.

### 2.5. Measurement of AGEs [2]

#### 2.5.1. Chemicals and Reagents

Standards CML, CEL, and MG-H1 and internal standards CML-d_4_, CEL-d_4_, and MG-H1-d_3_ were purchased from PolyPeptide Group (Strasbourg, France). All other reagents were of the highest grade available and were purchased from Wako (Osaka, Japan).

Each stock solution of CML, CEL, and MG-H1 at 0.1 mg/mL was prepared in purified water and stored at −20°C. Stock solutions of internal standards CML-d_4_, CEL-d_4_, and MG-H1-d_3_, at 0.1 mg/mL, were prepared in the same manner. Before analysis, we prepared working standard solutions (final concentrations 0–20 ng/mL) and internal standard working solutions (final concentration 10 ng/mL) by diluting the stock solutions using 1% aqueous formic acid.

#### 2.5.2. Instruments

High-performance liquid chromatography experiments were performed on a prominence series liquid chromatograph system (Shimadzu, Kyoto, Japan) consisting of a binary pump, in-line degasser, autosampler, and column oven. Chromatographic separation was achieved with an Intrada Amino Acid column (2.0 mm I.D. × 150 mm, Imtakt Co. Ltd., Kyoto, Japan) at 40°C with an injection volume of 10 *μ*L. The mobile phase consisted of solvent A, containing 100 mM ammonium formate in water, and solvent B, containing 0.5% formic acid in acetonitrile. The separation conditions were a linear gradient from 75 to 50% of solvent B from 0 to 7 min, from 50 to 0% of solvent B from 7 to 9 min, and 0% of solvent B from 9 to 20 min. The flow rate was 0.3 mL/min. The column was equilibrated for 6 min under the initial conditions before each injection.

An AB Sciex QTRAP 5500 mass spectrometer (AB SCIEX, Tokyo, Japan) equipped with an electrospray ion source in the positive ion mode was used under the following operating conditions: curtain gas 10 psi; ion spray voltage 5000 V; temperature of ion source 700°C; ion source gas 1 50 psi; ion source gas 2 70 psi; collision gas 8.0 psi; and entrance potential 10 V. Seven glycation free adducts were detected individually in the postcolumn by MS/MS with multiple reaction monitoring (MRM) for transition of the parent ions to the product ions. LC-MS/MS data were acquired and processed using Analyst version 1.5 software (Applied Biosystems/MDS Analytical Technologies, Tokyo, Japan).

#### 2.5.3. Preparation of Serum Extracts

Whole blood was collected into vacuum blood collection tubes containing a serum separating agent and a procoagulant film (VENOJECT II, Terumo Co., Tokyo, Japan) and refrigerated at 4°C. After standing for 30 min, serum was obtained by centrifugation at 3000 ×g for 10 min and stored at −80°C until deproteinization. For deproteinization, an aliquot of serum (50 *μ*L) was mixed with 6% aqueous sulfosalicylic acid (50 *μ*L) and centrifuged at 13000 rpm for 5 min. The supernatant was transferred to a filter unit (ULTRAFREE-C3LCR, 0.2 *μ*m, Merck Millipore, Darmstadt, Germany) and centrifuged below 12000 ×g for 5 min. The supernatant was transferred to a microtube and then diluted three times with 1% aqueous formic acid, forming the serum extract.

#### 2.5.4. Method Validation

Matrix effects of the serum extracts were determined by preparing mixtures containing 25, 100, and 200 ng/mL of each AGE in 1% aqueous formic acid. Next, 5 *μ*L of each mixture was added to 45 *μ*L of a serum extract or 1% aqueous formic acid. For the blank sample (0 ng/mL), 5 *μ*L of water was added to 45 *μ*L of a serum extract or 1% aqueous formic acid. The final concentrations of the AGEs were 0, 2.5, 10, and 20 ng/mL. These standard solutions dissolved in serum extracts or 1% aqueous formic acid were stored at −30°C until LC-MS/MS analysis.

An internal standard method using isotopic AGEs was also performed. Aliquots of the serum extracts (45 *μ*L) were dispensed and spiked with a mixture of three internal standards (CML-d_4_, CEL-d_4_, and MG-H1-d_3_) at a final concentration of 10 ng/mL (5 *μ*L) and stored at −30°C until LC-MS/MS analysis. Calibration curves of analyte/internal standard peak area ratio versus AGE concentration were constructed for each of the three AGEs.

Samples were prepared for a recovery test by mixing 45 *μ*L of the serum extract with 5 *μ*L of the respective stock solutions (CEL, CML, and MG-H1; final concentration: 2.5, 10, and 20 ng/mL, resp.) containing a mixture of three internal standards (final concentration 10 ng/mL). Percent recovery was calculated according to the formula:

(1) $$\begin{array}{c} Rec\;\left(\%\right)=\left\{\frac{\left[C\left(a\right)-C\left(b\right)\right]}{C\left(c\right)}\right\}\times 100 \end{array}$$

where Rec is the recovery, *C*(a) is the concentration in spiked sample, *C*(b) is the initial concentration, and *C*(c) is the concentration of standard mixture of three AGEs.

Our methods about meal tolerance test, glucose clamp test, and AGE measurements were already described in the past reports [2, 13, 18].

#### 2.5.5. Statistical Analysis

Data are expressed as mean ± standard deviation of the mean. The area under the curve was calculated according to the trapezoidal rule. Differences in the mean value of AGEs between T2DM and non-DM participants were assessed using an unpaired *t*-test. Correlations between parametric clinical variables and AGEs were determined using Pearson's correlation analysis. Values of *P* < 0.05 were considered significant. SPSS software version 24.0 (SPSS, Chicago, IL, USA) was used for all analyses.

## 3. Results

When standard CML was analyzed by MS/MS with flow injection, CML showed an intense molecular ion at *m*/*z* 205.10 [M+H]+. Therefore, product ion scanning was conducted for the ion, and CML-specific fragment ions at *m*/*z* 84.10 [M+H−121]+ were identified. Similarly, MS/MS analysis was performed for CEL and MG-H1. The identified ions (*m*/*z* 219.10 and 84.10 for CEL, *m*/*z* 229.10 and 70.10 for MG-H1) allowed selective detection of analytes using electrospray ionization- (ESI-) MS with MRM. All of the settings for MRM are summarized in Table 2. Although the detailed information of the separation mode and sample preparation for the Intrada Amino Acid column has not been released by the supplier, the sample preparation method was suggested by the supplier, and separation and quantitation were very good for human serum samples the same as food samples [2]. Ion enhancement was found in CEL, CML, and MG-H1 when the serum was diluted to 6.67 times. However, it showed good sensitivity and linearity (*R*^2^ > 0.97) in the range of 0–20 ng/mL. Therefore, the concentration of CML, CEL, and MG-H1 was calculated from the peak area of AGE with the peak area of internal standard of CML-d4, CEL-d4, and MG-H1-d3.

The mean values of CEL and CML were significantly higher in T2DM participants than non-DM participants (Table 3). MG-H1 was also higher in T2DM participants than non-DM participants, but this difference was not statistically significant.

Across all T2DM participants, there were no correlations between the AGEs and HbA1c (Table 4). CML was strongly negatively correlated with the HOMA-%B and IRI area under the curve and showed a significant positive correlation with ISI. However, the AGEs did not correlate with GDR or HOMA-IR. CEL and did not correlate with any insulin secretion or resistance indexes.

Across all non-DM participants, CML, CEL, and MG-H1 did not correlate with HbA1c or any insulin secretion or resistance indexes (Table 4).

## 4. Discussion

This study shows that CML and CEL levels were significantly higher in T2DM than non-DM participants. CML was significantly negatively correlated with insulin secretion, HOMA-%B and IRI-AUC, and positively correlated with ISI, but was not correlated with GDR in T2DM. A previous study using cell culture and animal models demonstrated that exposure to excess AGEs activates pathways of *β*-cell damage, which can impair insulin secretion [4]. *β*-Cells exposed to AGEs displayed acute glucose-stimulated insulin secretory defects, mitochondrial abnormalities including excess superoxide generation, and reduced calcium flux. Another study suggested that AGE injections can initiate *β*-cell dysfunction in vivo [25]. A recent in vitro study also indicated that CML caused mitochondrial dysfunction and mitophagy in *β*-cells and that high levels of AGEs may induce *β*-cell dysfunction and impair insulin secretion ability [26]. A recent epidemiological study also showed that increased fasting CML levels may be predictive of type 2 diabetes development [27]. These results suggest that CML decreases insulin secretion and is important in the pathophysiology of impaired glucose metabolism.

In our study, CML correlated with ISI, an index which is greatly affected by insulin secretion ability. Conversely, CML and the other AGEs did not correlate with the GDR. It may be difficult for the findings to explain the reasons, we consider that HOMA-IR mainly reflects the insulin resistance of liver and ISI mainly reflects the insulin resistance of muscle [28]. However, ISI is greatly affected by the insulin secretion ability, and if the insulin secretion ability is decreased, ISI shows low insulin resistance [29]. We measured GDR using the glucose clamp method, which is a precise method for assessing insulin resistance of muscle [28]. Therefore, we consider the results that CML correlates with ACU-IRI and ISI, but not GDR and HOMA-IR, which means CML mainly affects insulin secretion ability rather than insulin resistance. Thus, we suggest that CML has a greater effect on insulin secretion ability than on insulin resistance. A recent study reported that a diet low in AGEs increased insulin sensitivity in healthy, overweight individuals [30]. Insulin sensitivity as evaluated by the glucose clamp method increased after a low-AGE diet and showed a tendency to decrease after a high-AGE diet. There was no difference in body weight or insulin secretion between these diet groups. The authors suggested restricting dietary AGE content as an effective strategy to decrease diabetes and cardiovascular disease risks in overweight individuals. Another study reported that a low-AGE diet ameliorates insulin resistance in obese people with the metabolic syndrome without necessitating a major reduction in adiposity [31]. However, these studies differ from our study in that their subjects did not have type 2 diabetes. We suggest that the results of animal and cell culture studies on AGE content and diabetes [4] are likely to be more relevant to the present study; however, further study is needed.

Our study had several limitations. The relatively small number of participants (total 35, T2DM 15, non-DM 20) and the difference in age and BMI between the T2DM and non-DM groups indicate that our results require confirmation with a larger study. Therefore, we decided to add “A Brief Report” in the title. Furthermore, the DM group was aged and obese compared to the control group; therefore, the elevated levels of CML and CEL may be due to aging and obesity. However, glucose clamp test is a very complicated method, and it is difficult to recruit the patients with poorly controlled diabetes without medications and older nondiabetic participants with obesity. We are currently conducting a larger study, the results of which we plan to publish in the future. MTT was used in our study as OGTTs are best avoided in patients with severe diabetes because of the risk of hyperglycemia. As IGI and ISI were developed from OGTTs, we propose that differences between consuming a test meal and a pure glucose load may also affect glucose and insulin levels. Despite these limitations, we think our study contributes to our understanding of the pathophysiology of type 2 diabetes.

In summary, CML and CEL levels were significantly higher in T2DM than non-DM participants. CML was significantly negatively correlated with insulin secretion, HOMA-%B, and IRI-AUC and positively correlated with ISI in T2DM participants but was not correlated with insulin resistance as evaluated by the glucose clamp method. In conclusion, these results suggest that AGE might play a role in the development or prediction of insulin secretory defects in type 2 diabetes.

## Figures and Tables

**Table 1:** Participant characteristics.

	T2DM	Non-DM	*P* value
*n*	15	20	
Sex (male/female)	9/6	12/8	
Age (years)	56.1 ± 12.0	33.9 ± 9.5	<0.001
BMI (kg/m^2^)	27.46 ± 4.1	21.8 ± 2.9	<0.001
Waist circumference (cm)	95.7 ± 12.3	77.2 ± 9.9	<0.001
Fasting plasma glucose (mmol/L)	6.83 ± 0.75	4.80 ± 0.43	<0.001
HbA1c (%)	7.33 ± 0.83	5.33 ± 0.27	<0.001
HbA1c (mmol/mol)	(56.6)	(35.0)	
Insulinogenic index (IGI)	0.83 ± 1.07	1.44 ± 1.34	0.14
IRI-AUC	861.8 ± 429.2	616.3 ± 293.7	0.07
HOMA-%B (%)	73.7 ± 37.9	124.1 ± 68.8	<0.05
HOMA-IR	3.94 ± 2.50	1.70 ± 1.13	<0.001
Insulin sensitivity index (ISI)	4.11 ± 3.16	7.31 ± 3.32	<0.001
GDR	5.44 ± 2.34	9.52 ± 2.61	<0.001

Data are mean ± standard deviation. GDR: glucose disposal rate; HOMA-%B: homeostasis model assessment of beta cell function; HOMA-IR: homeostasis model assessment for insulin resistance; IRI-AUC: immunoreactive insulin area under the curve; Non-DM: nondiabetic study participants; T2DM: study participants with type 2 diabetes mellitus.

**Table 2:** Operating parameters for serum AGE measurement using a QTRAP 5500 mass spectrometer equipped with an electrospray ion source operating in the positive ion mode.

Compound	Retention time (min)	Precursor ion (*m/z*)	Product ion (*m/z*)	DP (V)	CE (V)	CXP (V)
CEL	5.64	219.1	84.1	111	27	6
CML	6.01	205.1	84.1	76	25	10
MG-H1	9.09	229.1	70.1	46	30	8

CEL-d_4_	5.65	223.1	88.1	111	27	6
CML-d_4_	5.99	209.1	88.1	76	25	10
MG-H1-d_3_	9.07	232.1	70.1	46	30	8

DP: declustering potential; CE: collision energy; CXP: collision cell exit potential; CEL: carboxyethyllysine; CML: carboxymethyllysine; MG-H1: methyl-glyoxal-hydro-imidazolone.

**Table 3:** Serum AGEs levels.

AGE			
	CEL (ng/mL)	CML (ng/mL)	MG-H1 (ng/mL)
T2DM (*n* = 15)	15.4 ± 6.6	21.0 ± 7.0	27.2 ± 20.7
Non-DM (*n* = 20)	9.8 ± 2.2	15.1 ± 4.2	17.5 ± 8.2
*P* value	<0.001	<0.01	NS

Data are mean ± standard deviation. AGEs: advanced glycation end products; CEL: carboxyethyllysine; CML: carboxymethyllysine; MG-H1: methyl-glyoxal-hydro-imidazolone; T2DM: study participants with type 2 diabetes mellitus; Non-DM: nondiabetic study participants; NS: not significant.

**Table 4:** Correlation coefficients for the associations between AGEs and clinical parameters.

T2DM (*n* = 15)						
Index	CEL		CML		MG-H1	
	*r*	*P*	*r*	*P*	*r*	*P*

HbA1c	−0.43	NS	−0.10	NS	−0.11	NS
IGI	−0.31	NS	−0.39	NS	−0.30	NS
IRI-AUC	−0.27	NS	−0.53	<0.05	−0.20	NS
HOMA-%B	−0.14	NS	−0.63	<0.05	0.01	NS
HOMA-IR	−0.13	NS	−0.47	NS	−0.04	NS
ISI	0.26	NS	0.72	<0.01	−0.04	NS
GDR	0.31	NS	0.13	NS	0.15	NS

Non-DM (*n* = 20)						
Index	CEL		CML		MG-H1	
	*r*	*P*	*r*	*P*	*r*	*P*

HbA1c	−0.03	NS	−0.10	NS	−0.05	NS
IGI	0.14	NS	0.26	NS	−0.03	NS
IRI-AUC	0.10	NS	0.24	NS	−0.18	NS
HOMA-%B	0.11	NS	0.05	NS	−0.29	NS
HOMA-IR	0.04	NS	−0.03	NS	−0.12	NS
ISI	−0.12	NS	−0.24	NS	−0.12	NS
GDR	0.16	NS	0.12	NS	0.03	NS

Correlation coefficients were determined using Pearson's product moment correlation coefficient test. AGEs: advanced glycation end products; CEL: carboxyethyllysine; CML: carboxymethyllysine; GDR: glucose disposal rate; HOMA-%B: homeostasis model assessment of beta cell function; HOMA-IR: homeostasis model assessment for insulin resistance; IGI: insulinogenic index; IRI-AUC: immunoreactive insulin area under the curve; ISI: insulin sensitivity index; MG-H1: methyl-glyoxal-hydro-imidazolone; Non-DM: nondiabetic study participants; T2DM: study participants with type 2 diabetes mellitus; NS: not significant.

## Abbreviations

AGEs: Advanced glycation end products
AUC: Area under the curve
CEL: Carboxyethyllysine
CML: Carboxymethyllysine
GDR: Glucose disposal rate
HOMA-%B: Homeostasis model assessment of beta cell function
HOMA-IR: Homeostasis model assessment for insulin resistance
IGI: Insulinogenic index
IRI: Immunoreactive insulin
ISI: Insulin sensitivity index
LC-MS/MS: Liquid chromatography-tandem mass spectrometry
MG-H1: Methyl-glyoxal-hydro-imidazolone
MTT: Meal tolerance test
Non-DM: Nondiabetic study participants
OGTT: Oral glucose tolerance test
T2DM: Study participants with type 2 diabetes mellitus.
